# Supplementary material for: Phenome-wide analyses establish a specific association between aortic valve PALMD expression and calcific aortic valve stenosis
Source: Commun Biol. 2020 Aug 28;3:477. doi: 10.1038/s42003-020-01210-x (PMC7455695; doi:10.1038/s42003-020-01210-x)
Supplement: Supplementary file 1 — Supplementary Information [file 42003_2020_1210_MOESM1_ESM.pdf]

SUPPLEMENTARY INFORMATION

Phenome-wide analyses establish a specific association between aortic valve PALMD expression and calcific aortic valve stenosis

Supplementary Figure 1. Phenome-wide association study of rs6702619-G in the UK Biobank

Supplementary Figure 2. Phenome-wide association studies of *PALMD* genetically-determined expression in 8 tissues in the UK Biobank

Supplementary Figure 3: Relationship between *PALMD* eQTL in 8 tissues and GWAS association with CAVS

Supplementary Figure 4. Relationship between *PALMD* eQTL in selected tissues and GWAS association with Atrial fibrillation

Supplementary Table 1. Association between *PALMD* genetically-determined expression in the aortic valve and CAVS in the UK Biobank and QUEBEC-CAVS

Supplementary Table 2. Description of the public GWAS meta-analysis consortia of cardiovascular traits used to evaluate the impact of *PALMD* predicted expression in various tissues

Supplementary Figures

Supplementary Figure 1. Phenome-wide association study of rs6702619-G in the UK Biobank

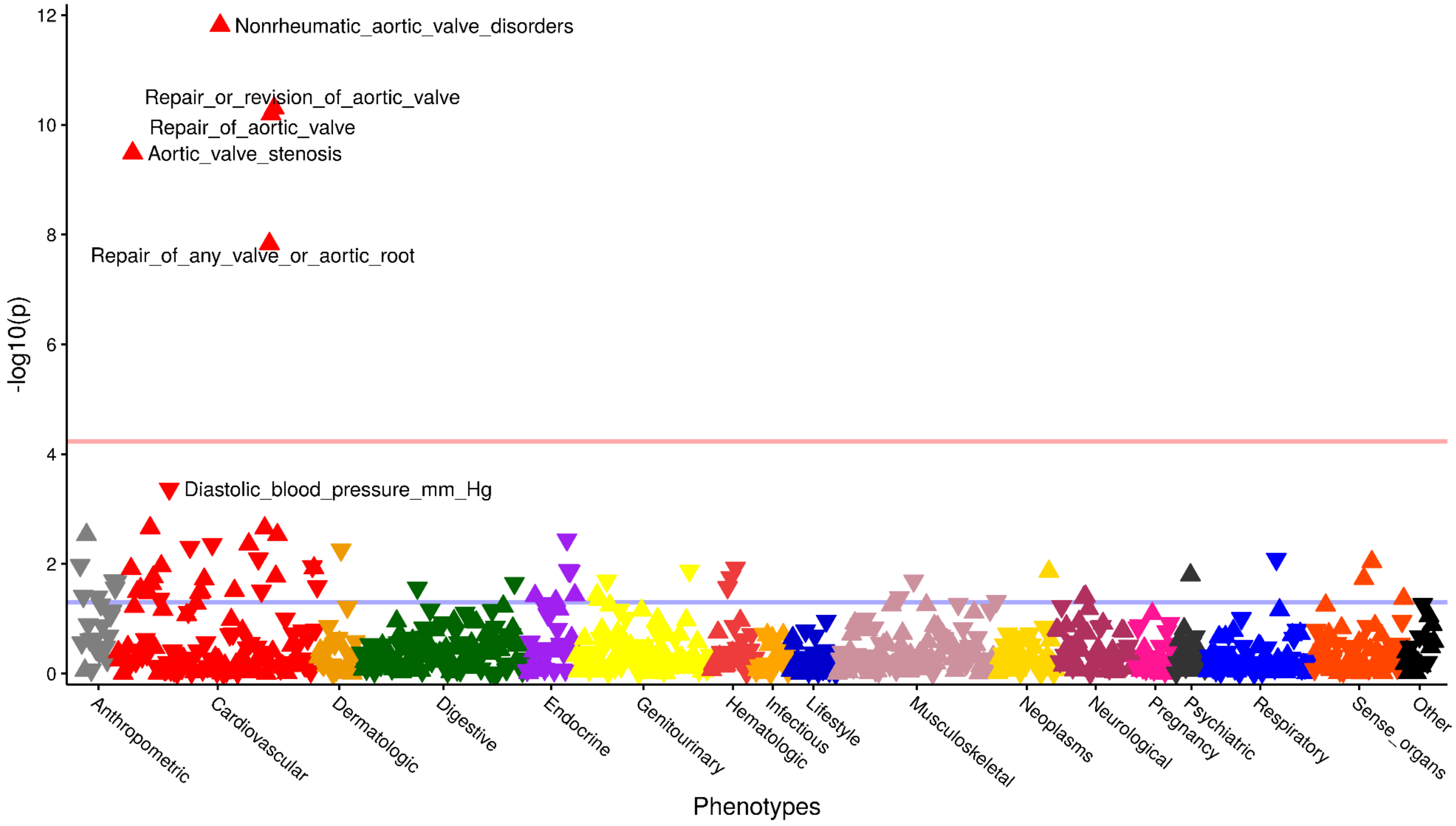

Each triangle represents a different phenotype (n=852). Triangles pointing up and down are positive and negative associations with rs6702619-G, respectively. The pink horizontal line represents the threshold for significance after correcting for multiple testing (P=0.05/852=5.9×10^{-5}). The blue horizontal line represents the threshold for nominal significance (P=0.05).

Supplementary Figure 2. Phenome-wide association studies of *PALMD* genetically-determined expression in 8 tissues in the UK Biobank

A) Brain anterior cingulate cortex

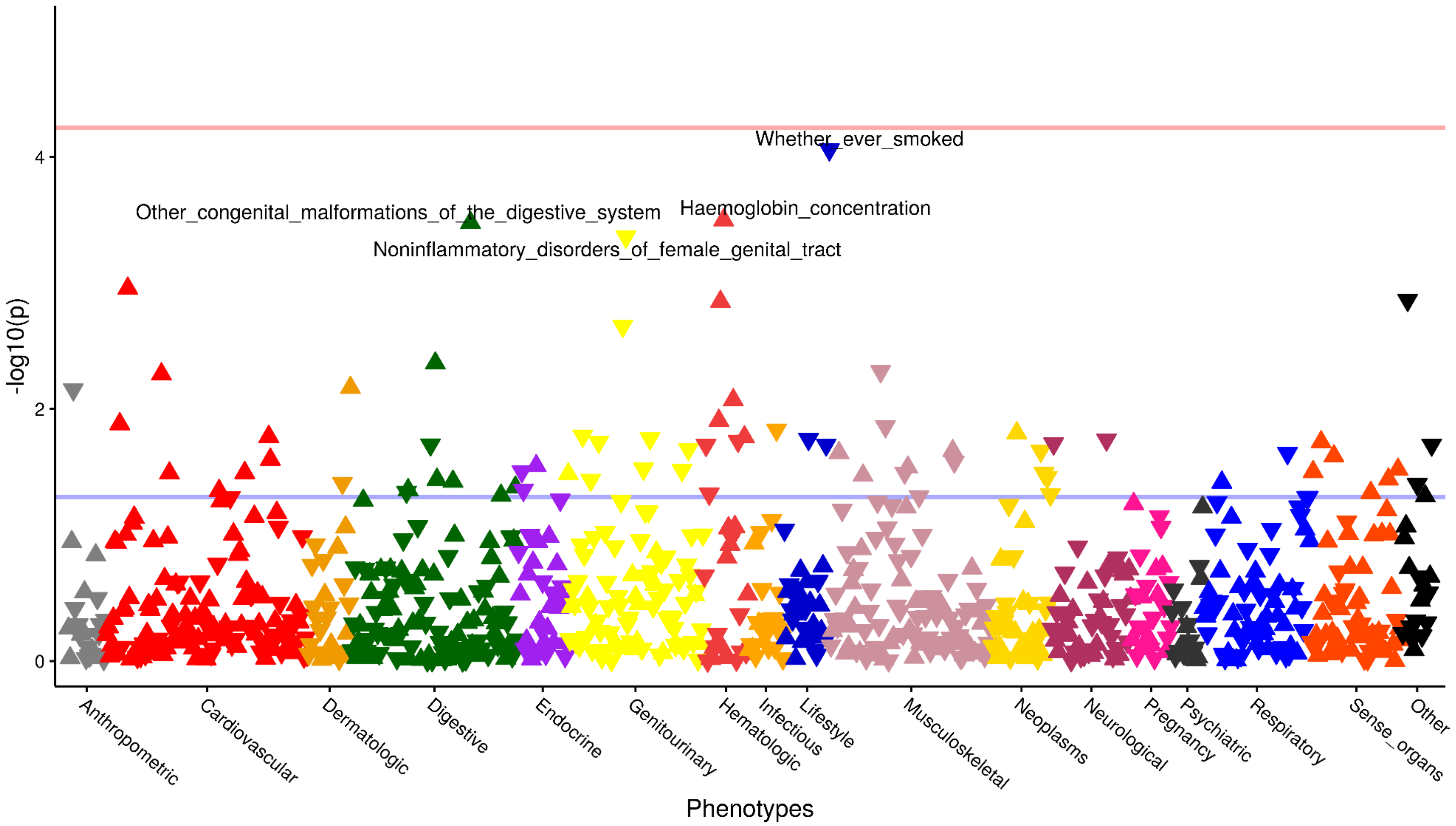

B) Transformed fibroblasts

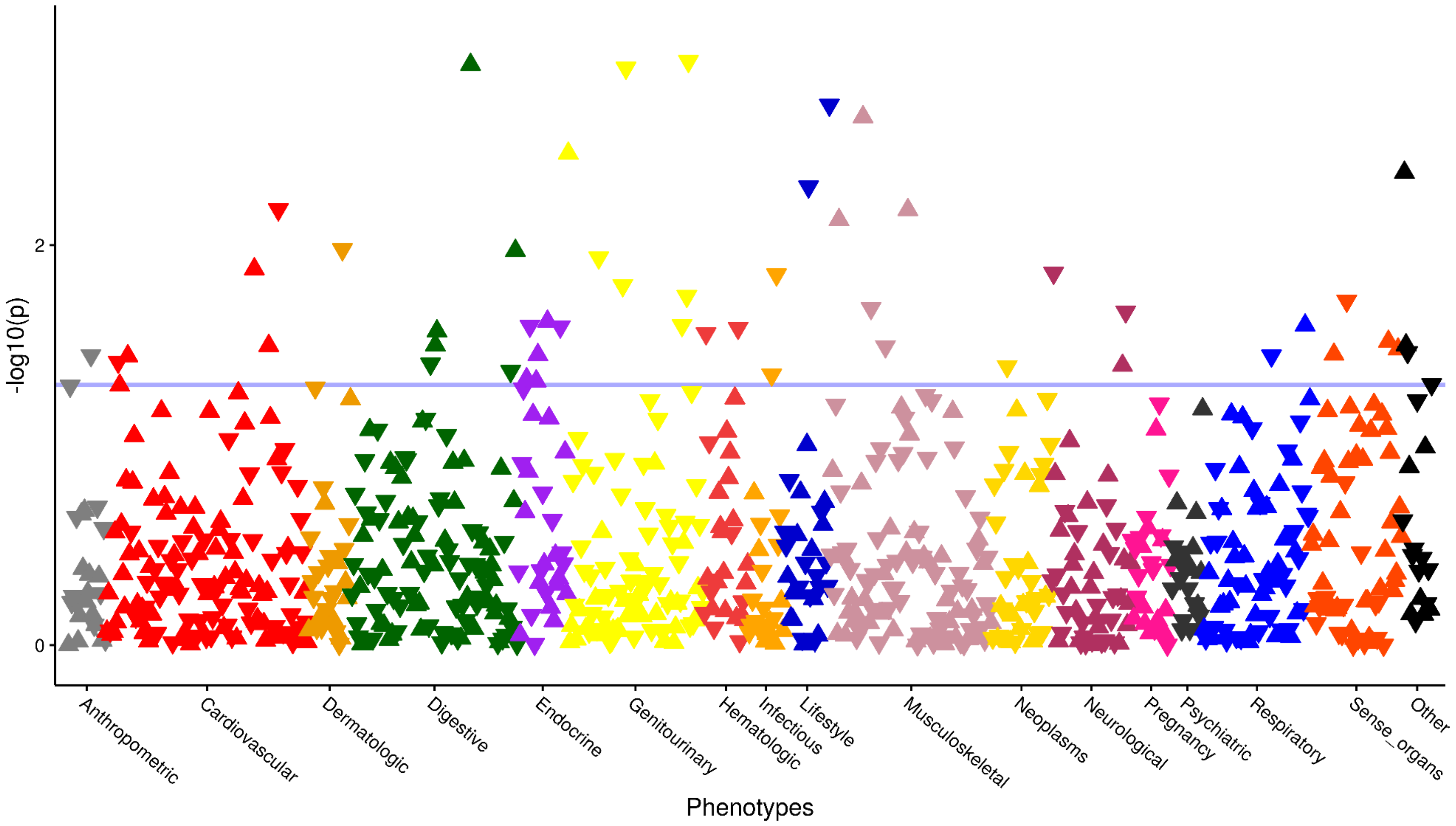

C) Gastroesophageal junction

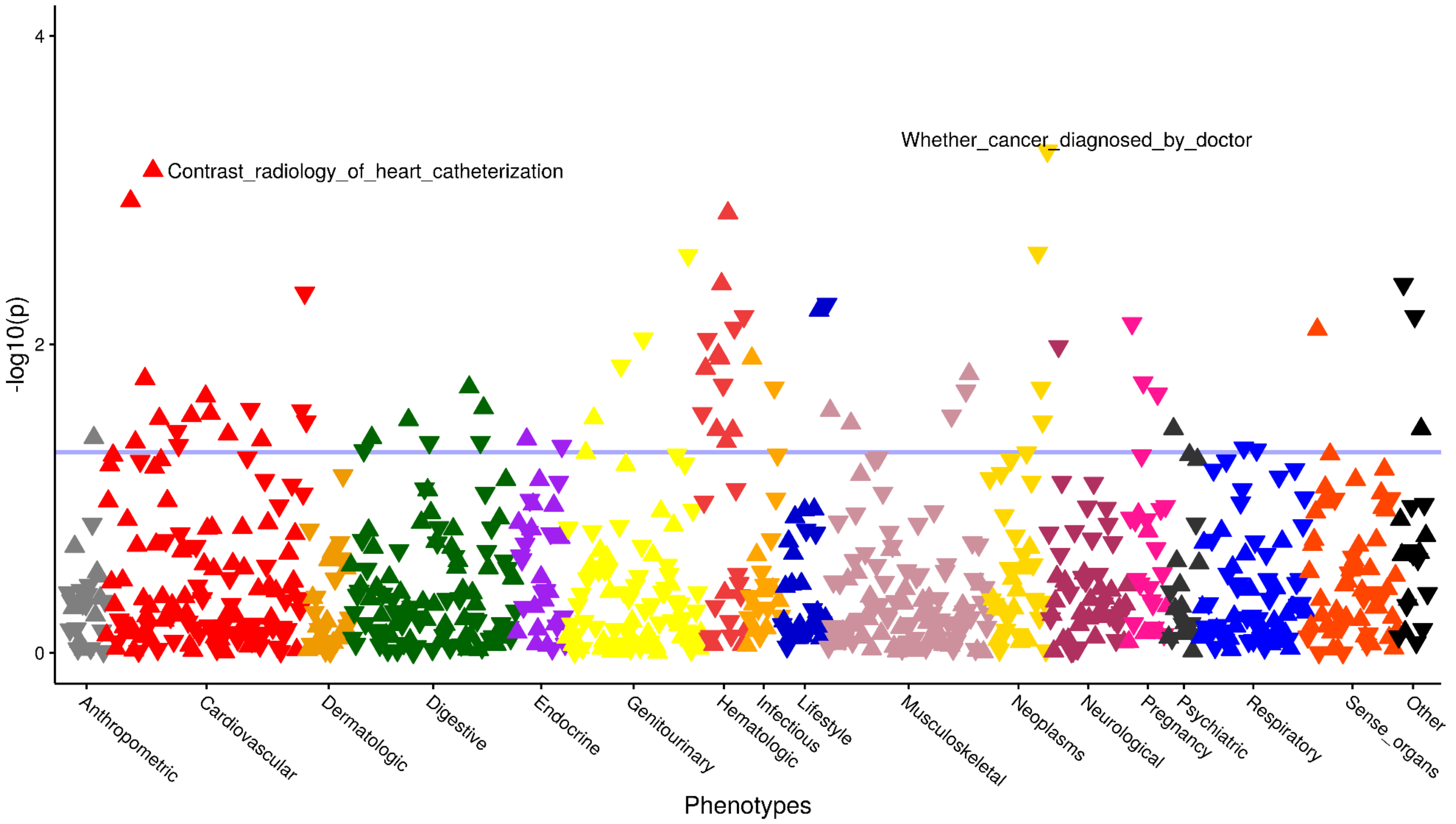

D) Esophagus mucosa

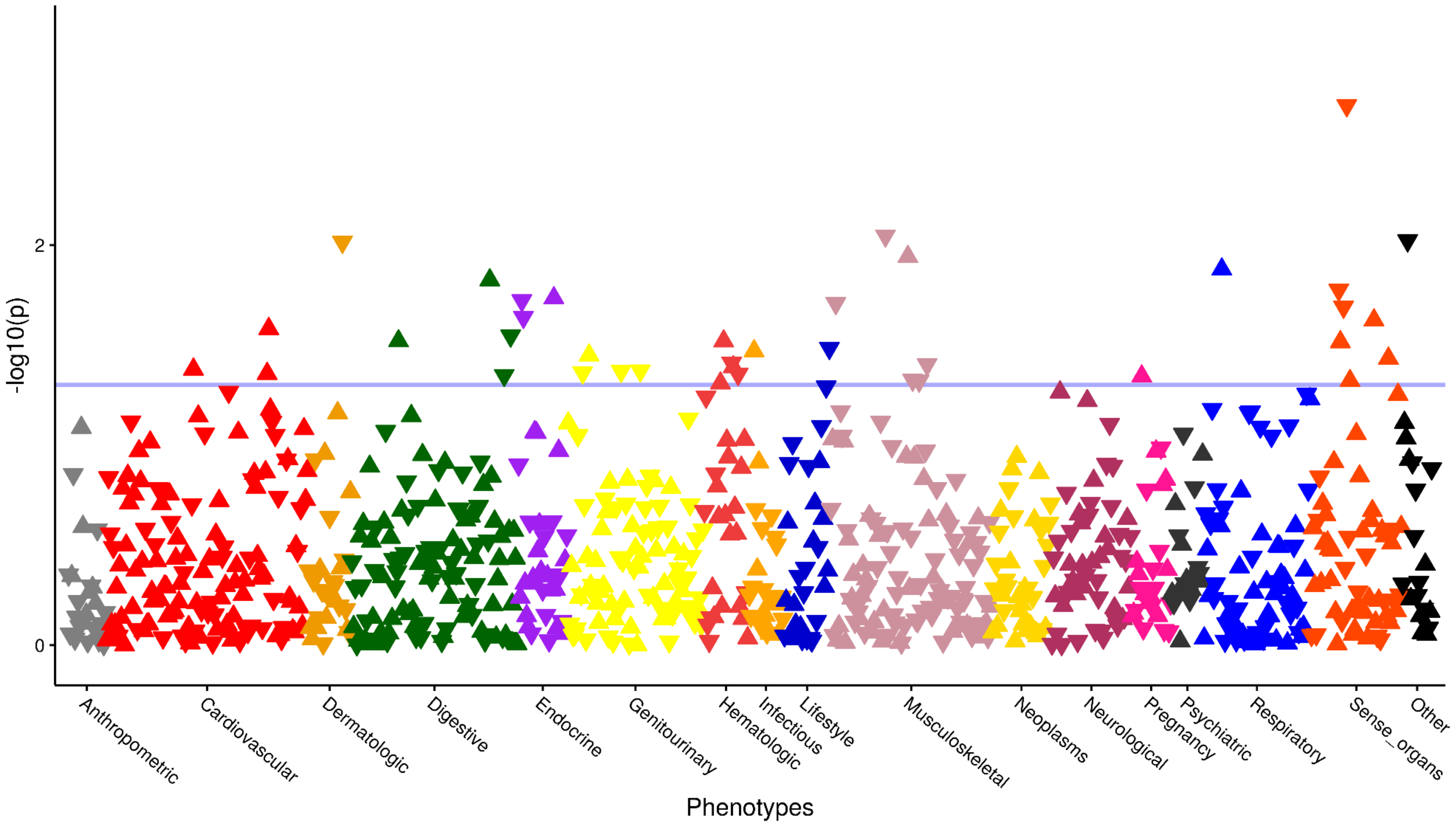

E) Esophagus muscularis

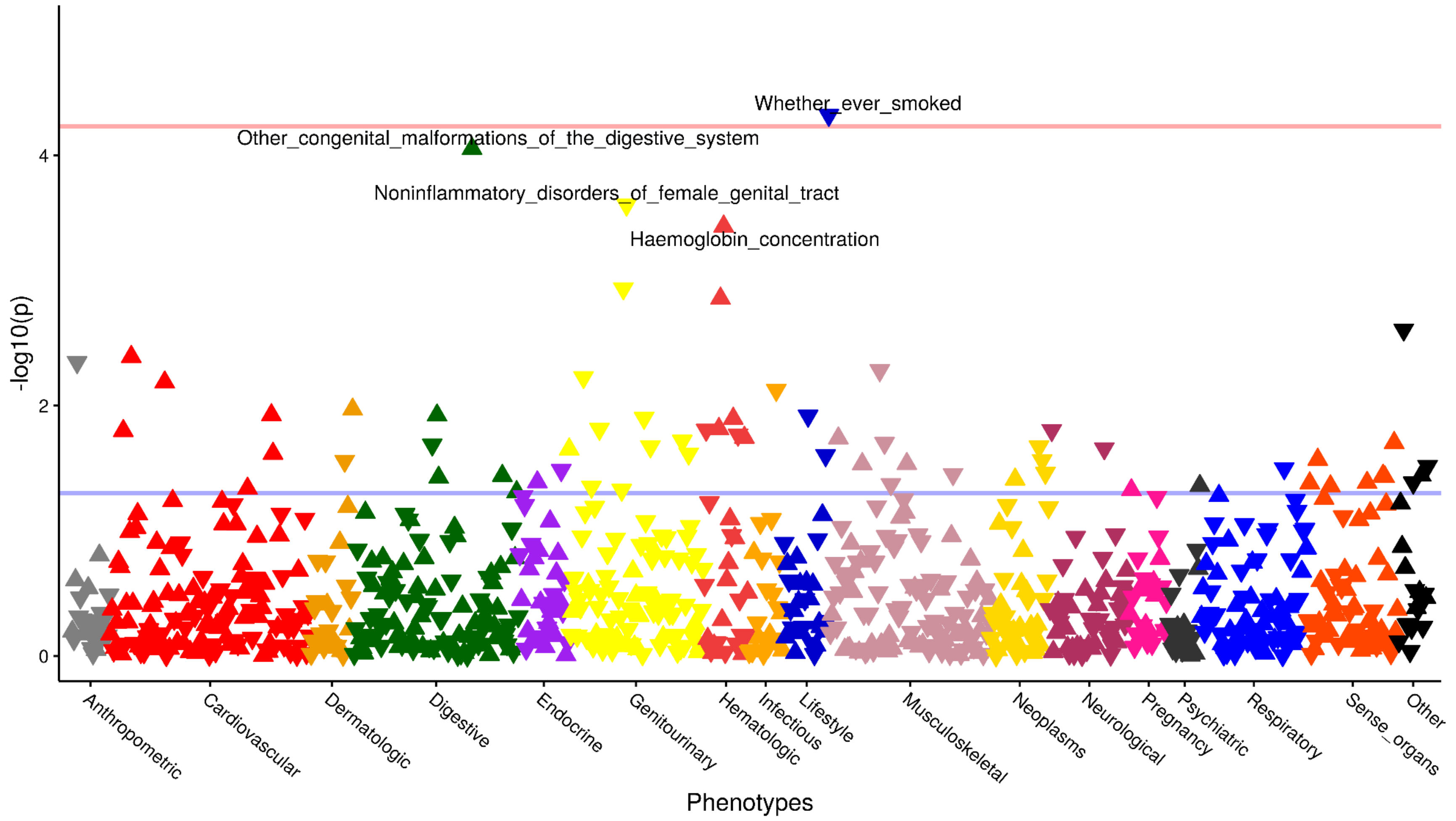

F) Tibial nerve

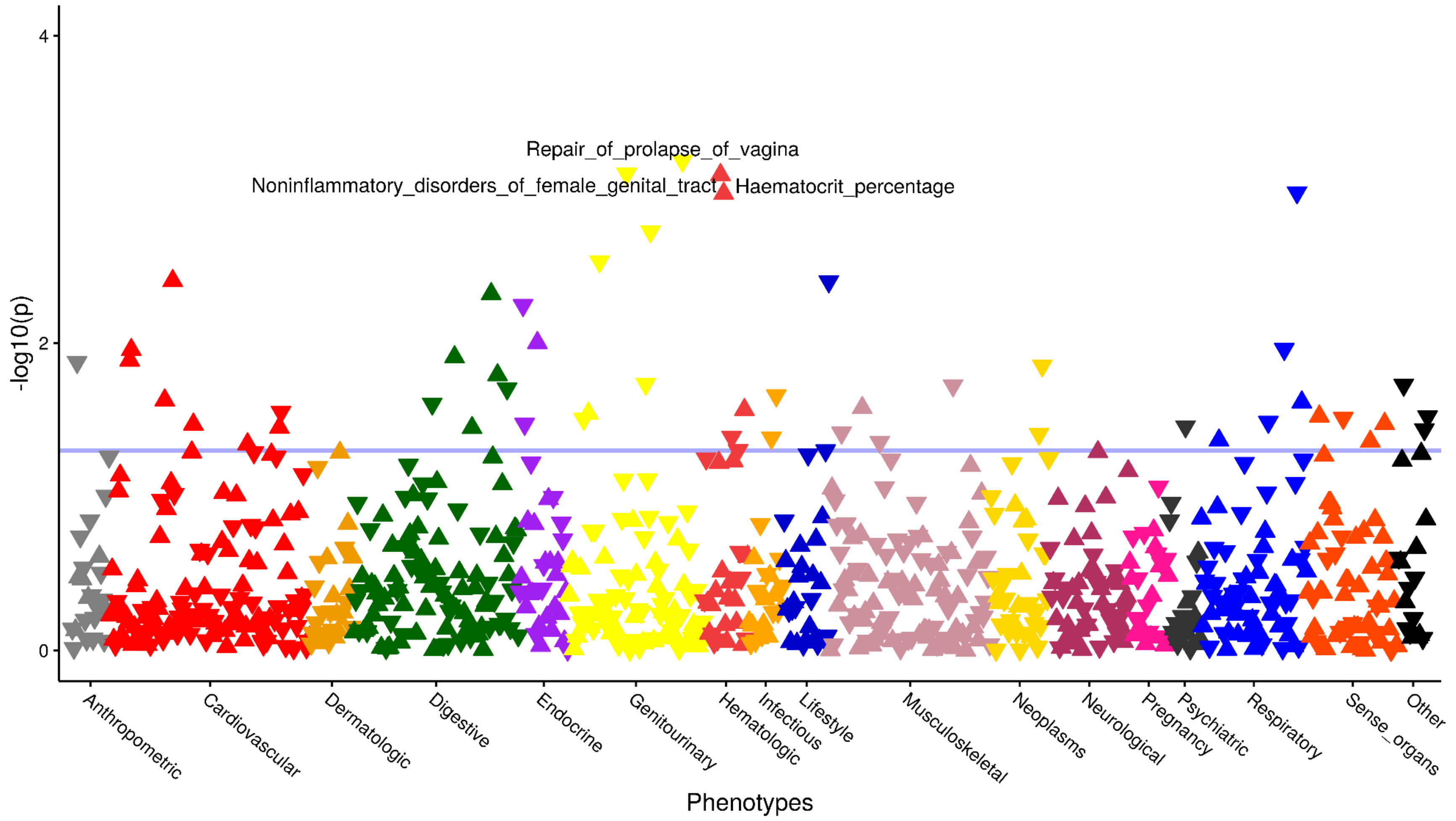

G) Pancreas

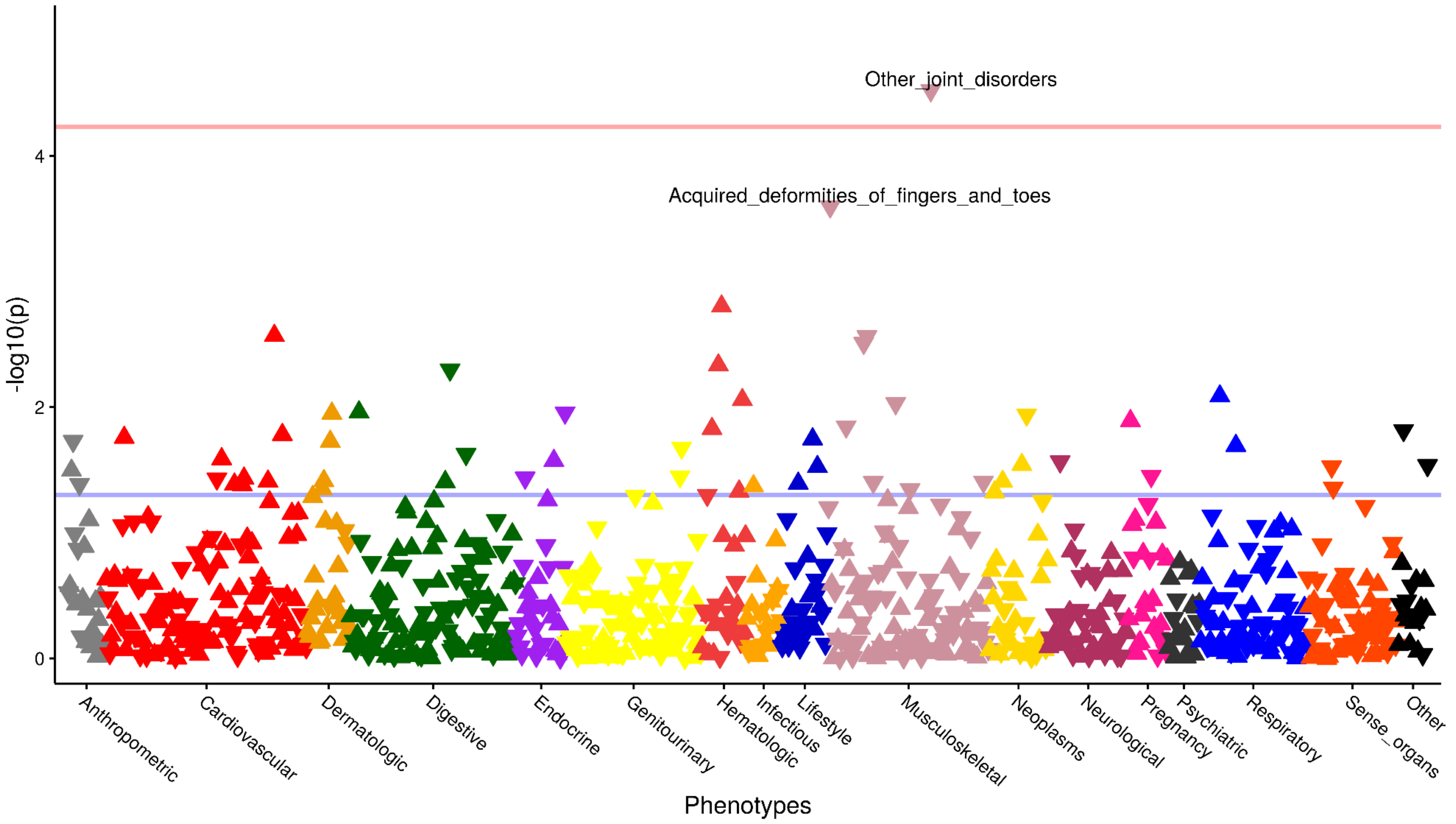
Other_joint_disorders
Acquired_deformities_of_fingers_and_toes
-log10(p)
4
2
0
Anthropometric
Cardiovascular
Dermatologic
Digestive
Endocrine
Genitourinary
Hematologic
Infectious
Lifestyle
Musculoskeletal
Neoplasms
Neurological
Pregnancy
Psychiatric
Respiratory
Sense_organs
Other
Phenotypes

H) Subcutaneous adipose tissue

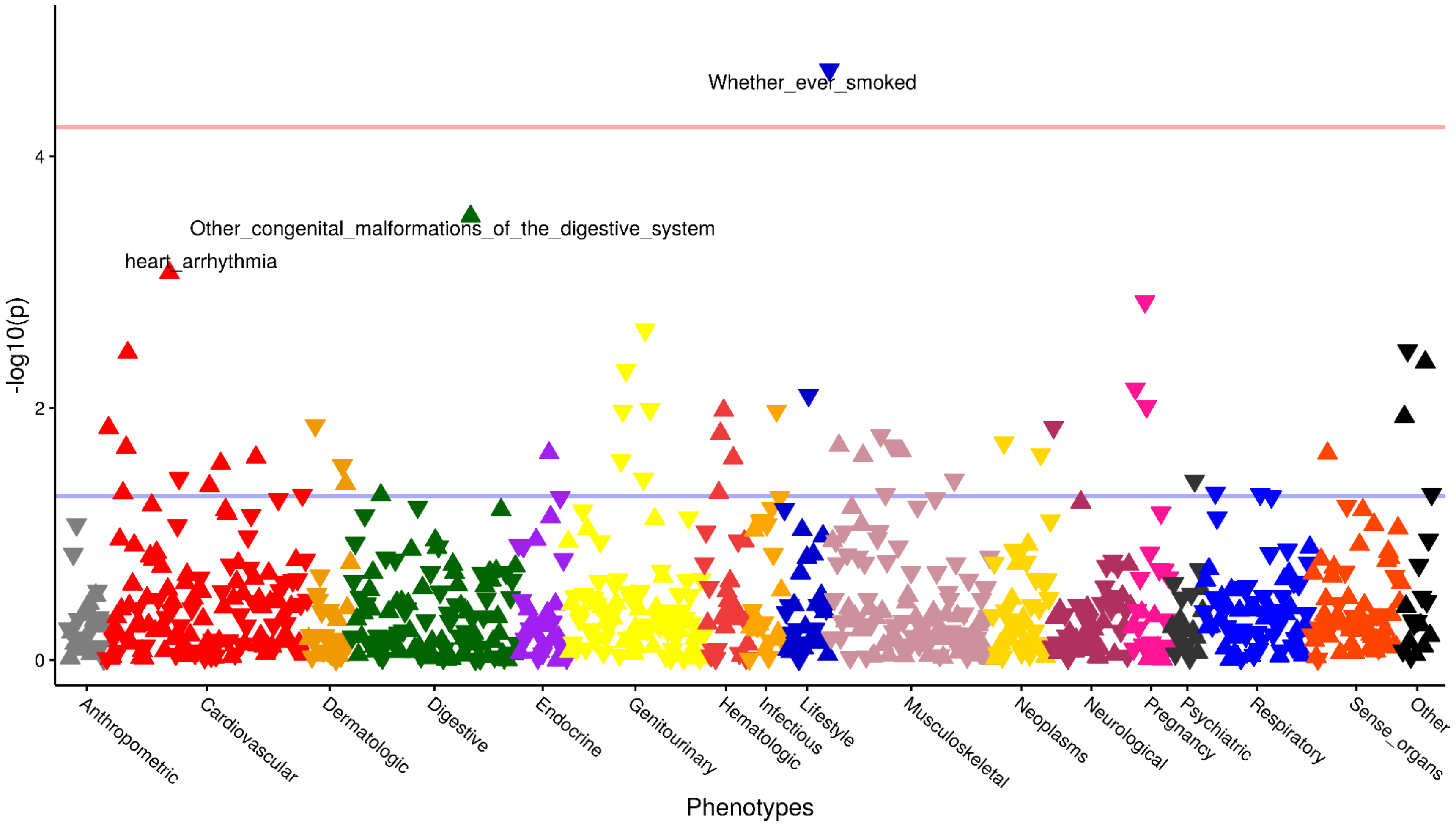

Phenome-wide association study of *PALMD* genetically-determined expression in **A** Brain anterior cingulate cortex; **B** Transformed fibroblasts; **C** Gastroesophageal junction; **D** Esophagus mucosa; **E** Esophagus muscularis; **F** Tibial nerve; **G** Pancreas; **H** Subcutaneous adipose tissue. Each triangle represents a different phenotype (n=852). Triangles pointing up and down are positive and negative associations with *PALMD* genetically-determined expression in the respective tissue. The pink horizontal line represents $P=0.05/852=5.9\times10^{-5}$. The blue horizontal line represents the threshold for nominal significance ($P=0.05$).

Supplementary Figure 3. Relationship between *PALMD* eQTL in 8 tissues and GWAS association with CAVS

A) Brain anterior cingulate cortex

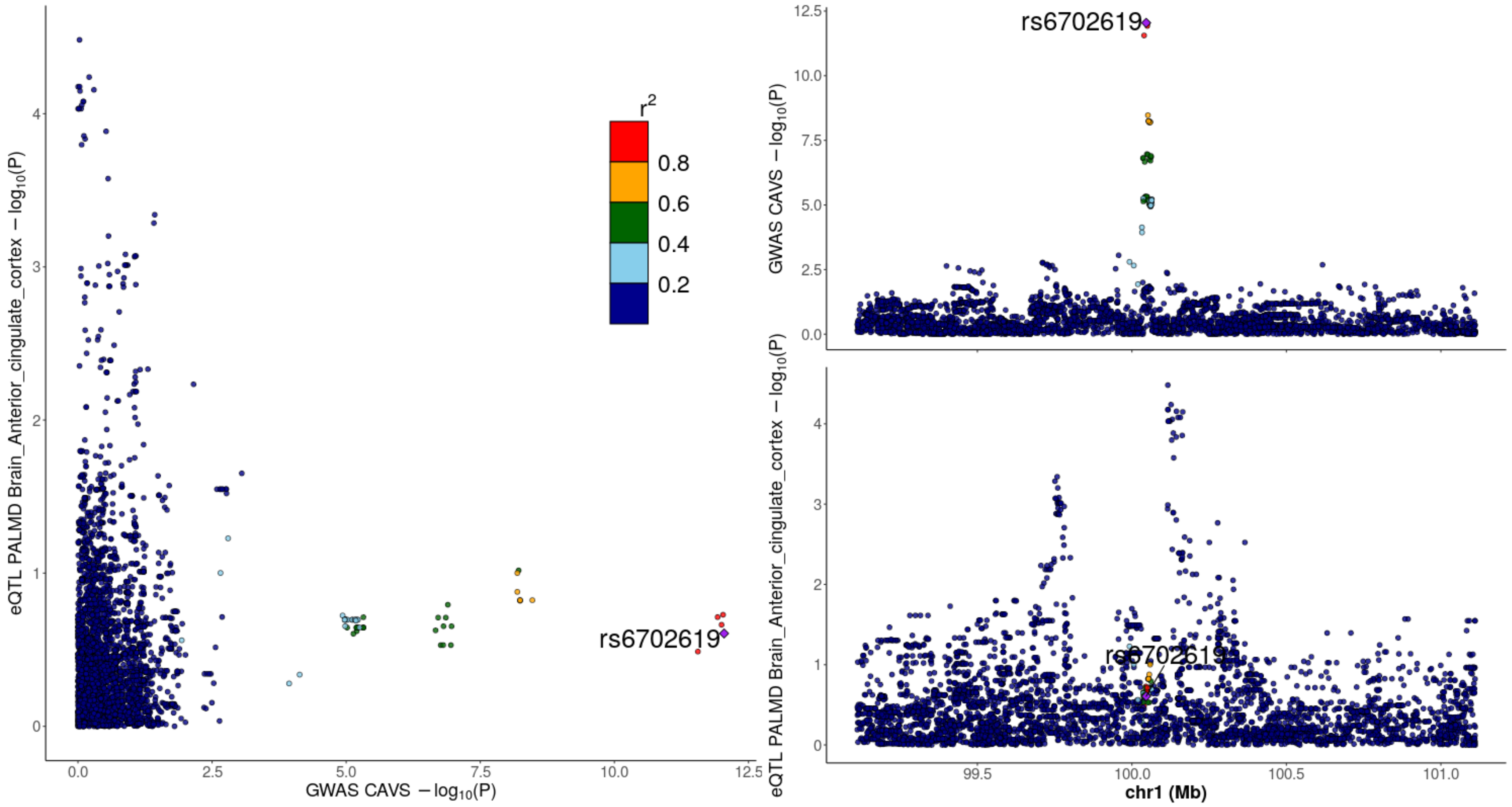

B) Transformed fibroblasts

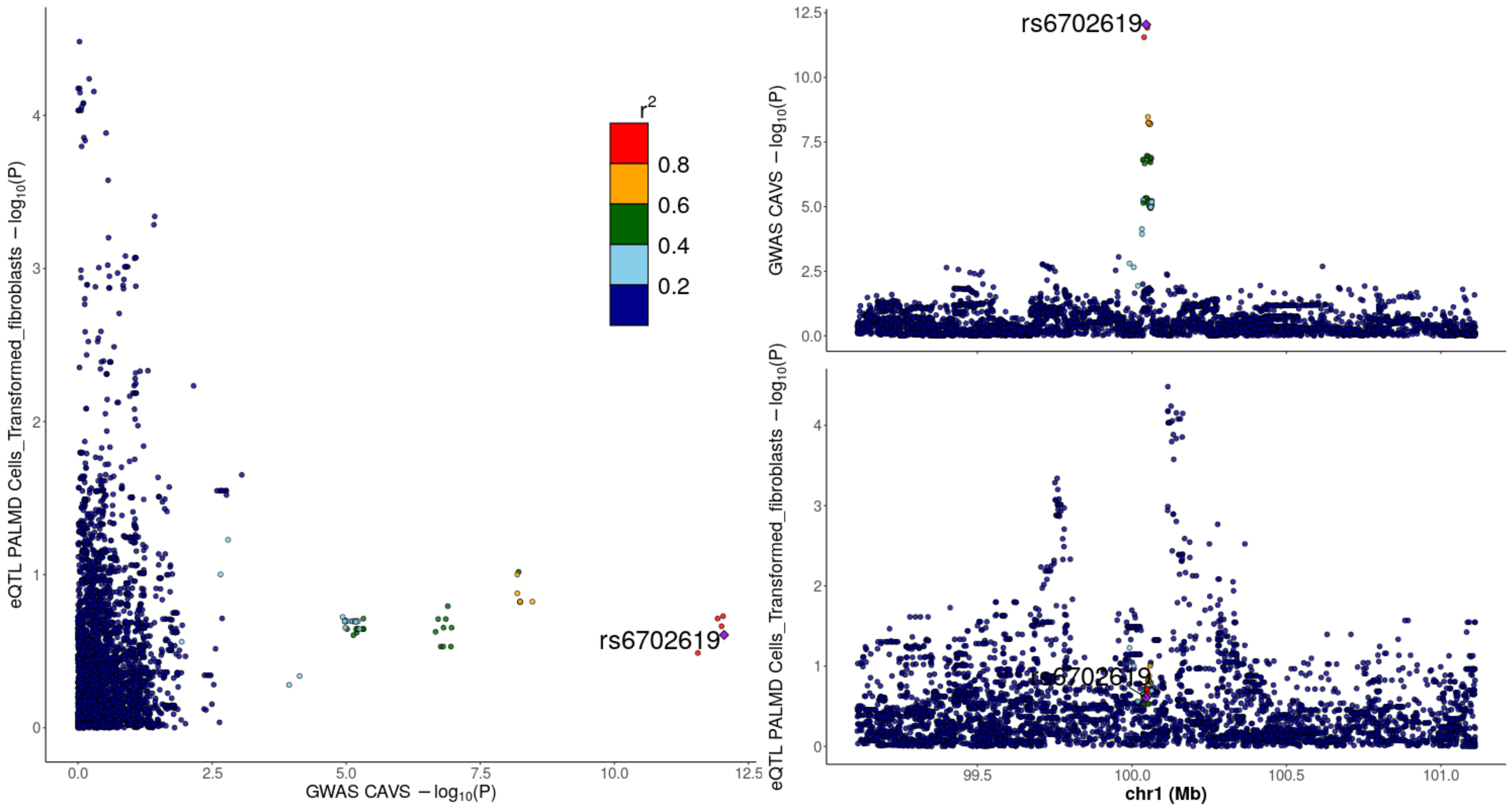

C) Gastroesophageal junction

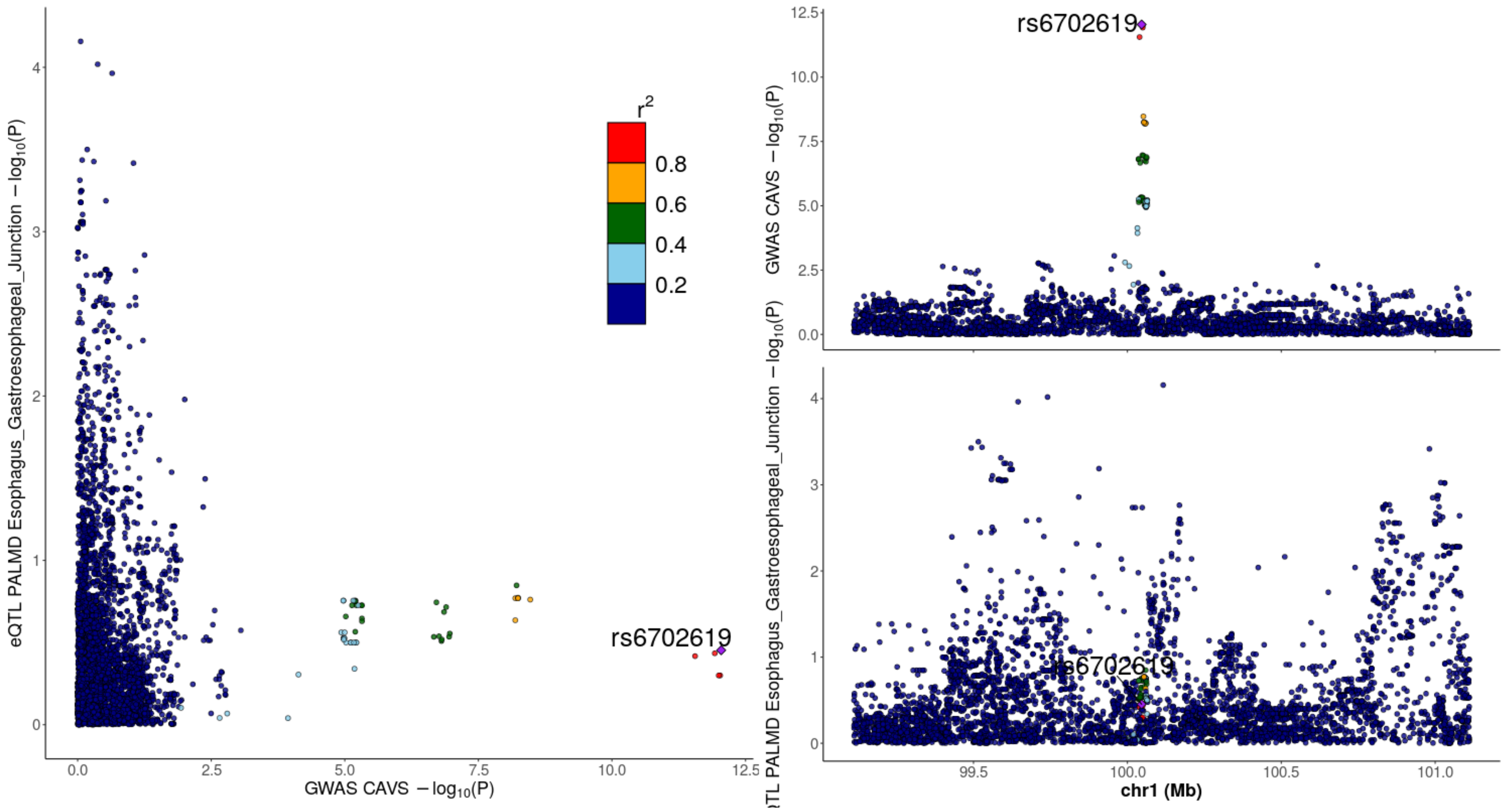

D) Esophagus mucosa

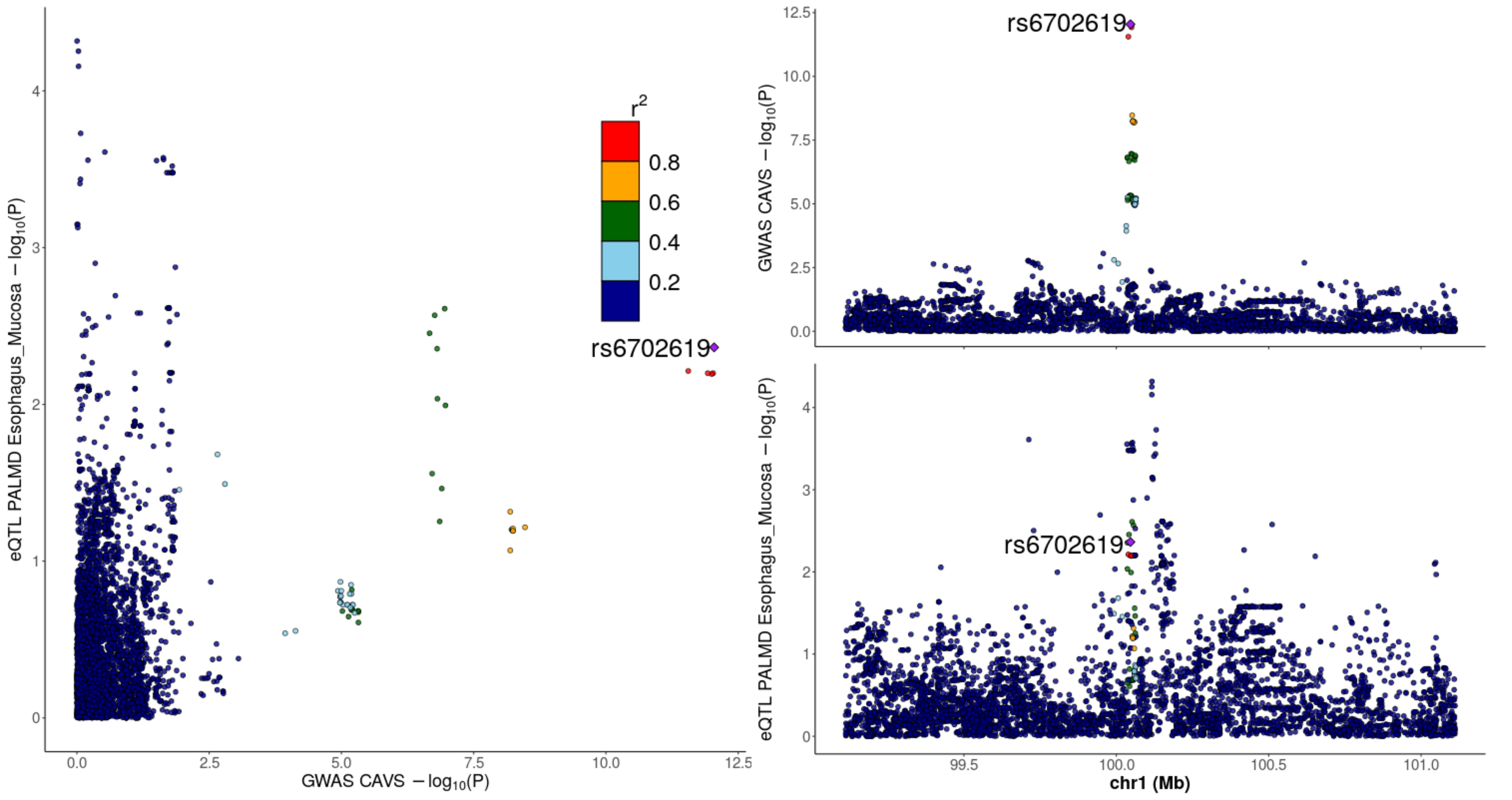

E) Esophagus muscularis

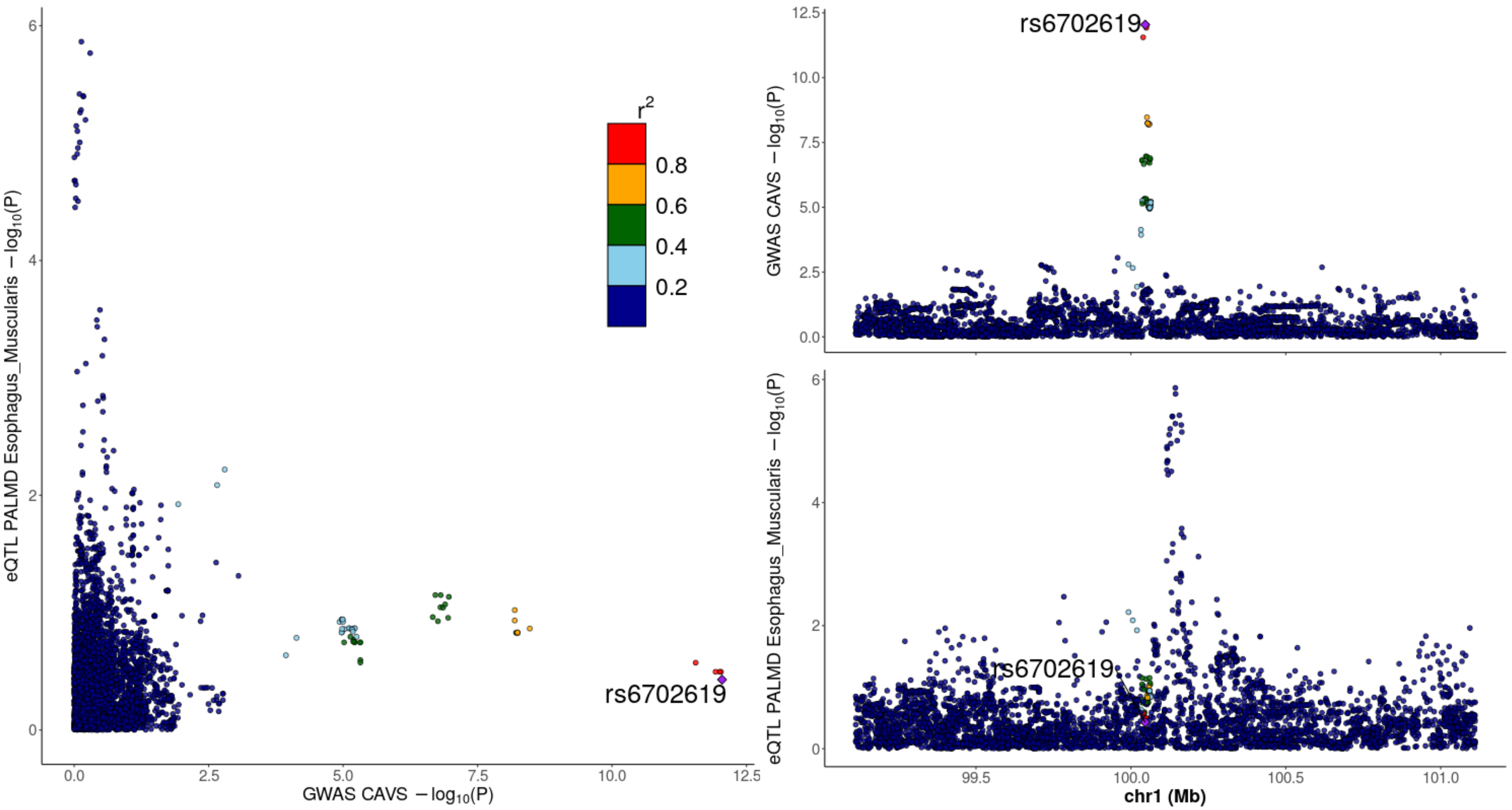

F) Tibial nerve

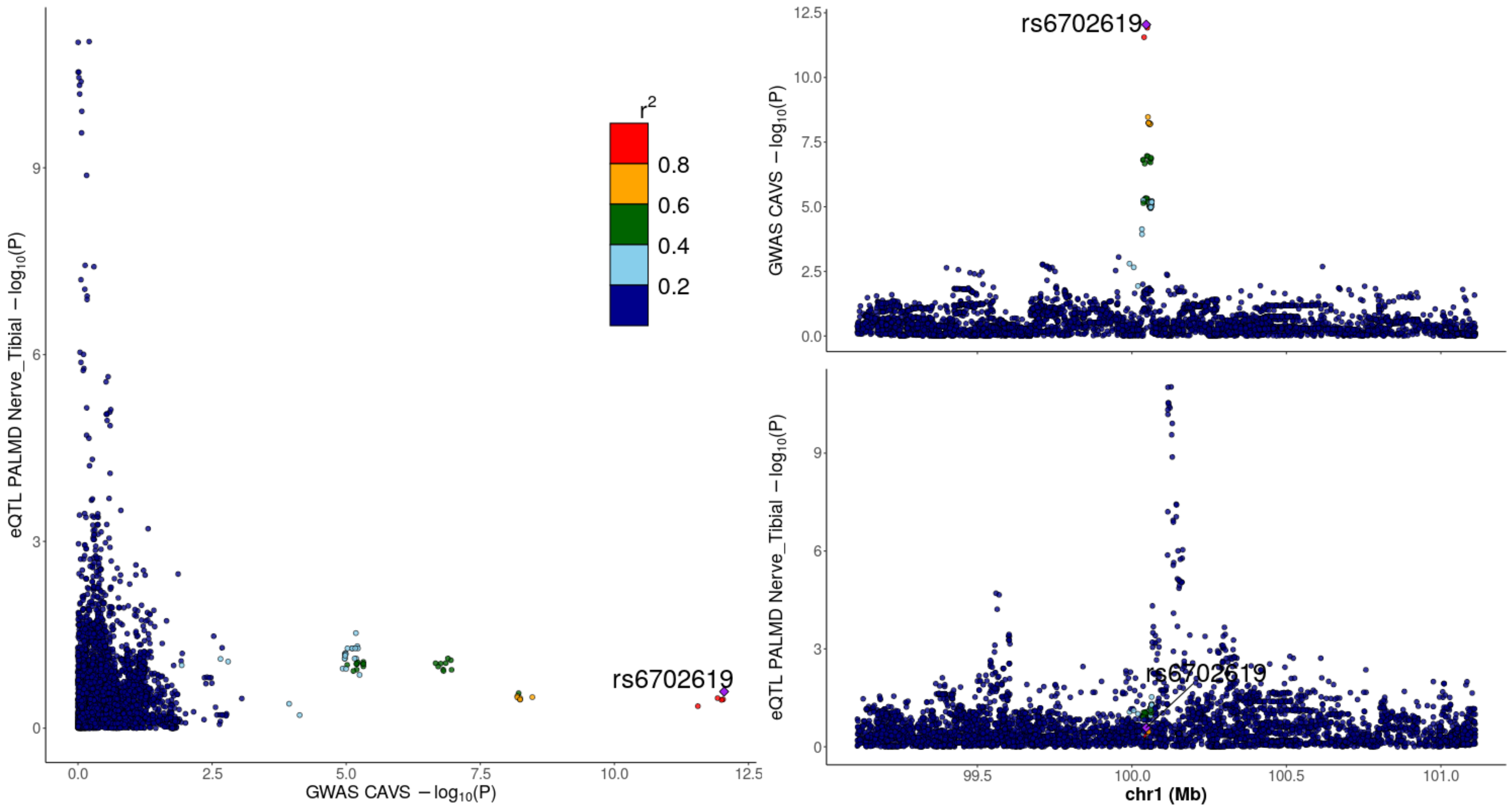

G) Pancreas

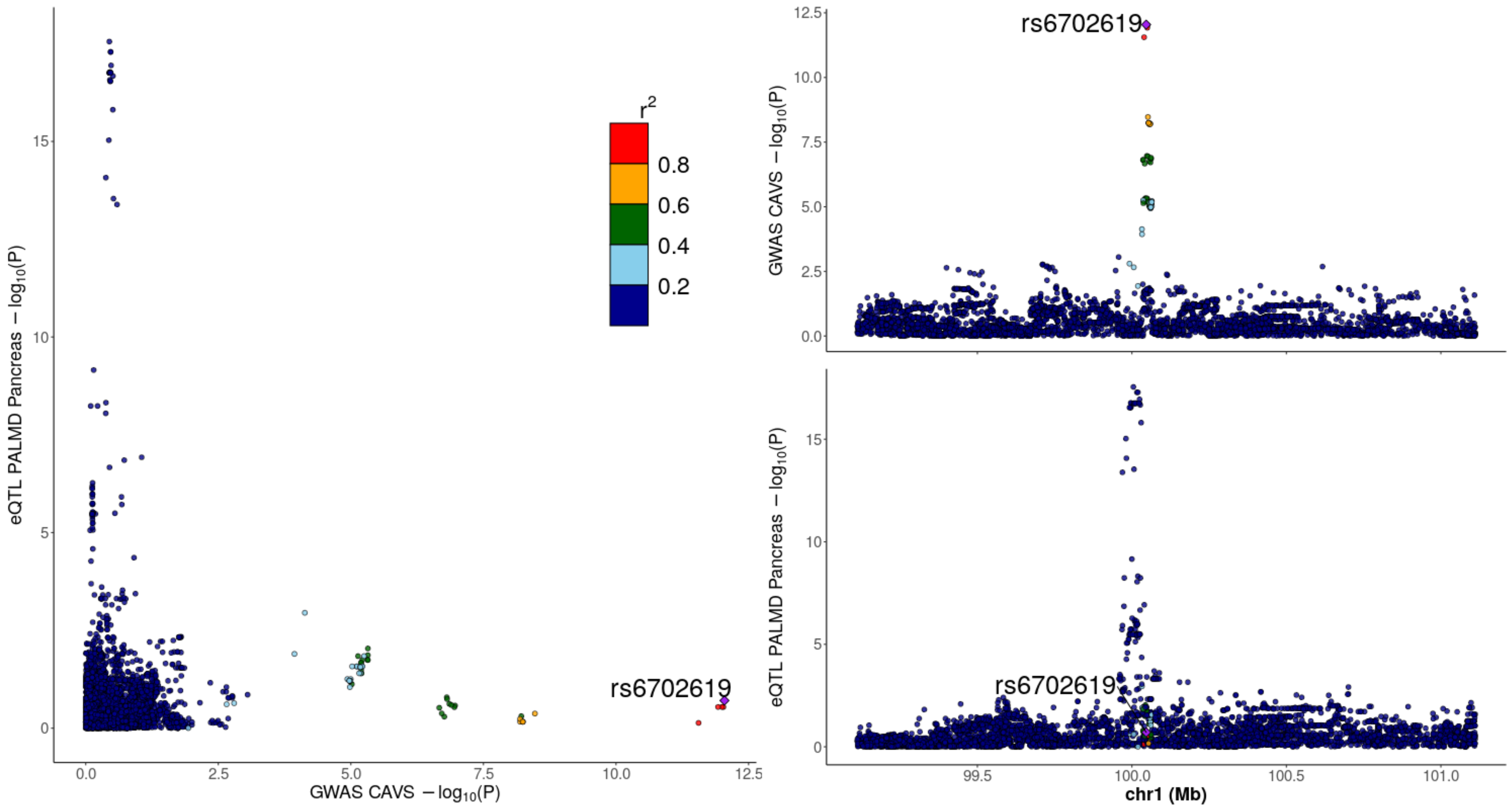

r²
0.8
0.6
0.4
0.2
rs6702619
eQTL PALMD Pancreas − log10(P)
GWAS CAVS − log10(P)
0
5
10
15
0.0
2.5
5.0
7.5
10.0
12.5
rs6702619
GWAS CAVS − log10(P)
0.0
2.5
5.0
7.5
10.0
12.5
rs6702619
eQTL PALMD Pancreas − log10(P)
0
5
10
15
99.5
100.0
100.5
101.0
chr1 (Mb)

H) Subcutaneous adipose tissue

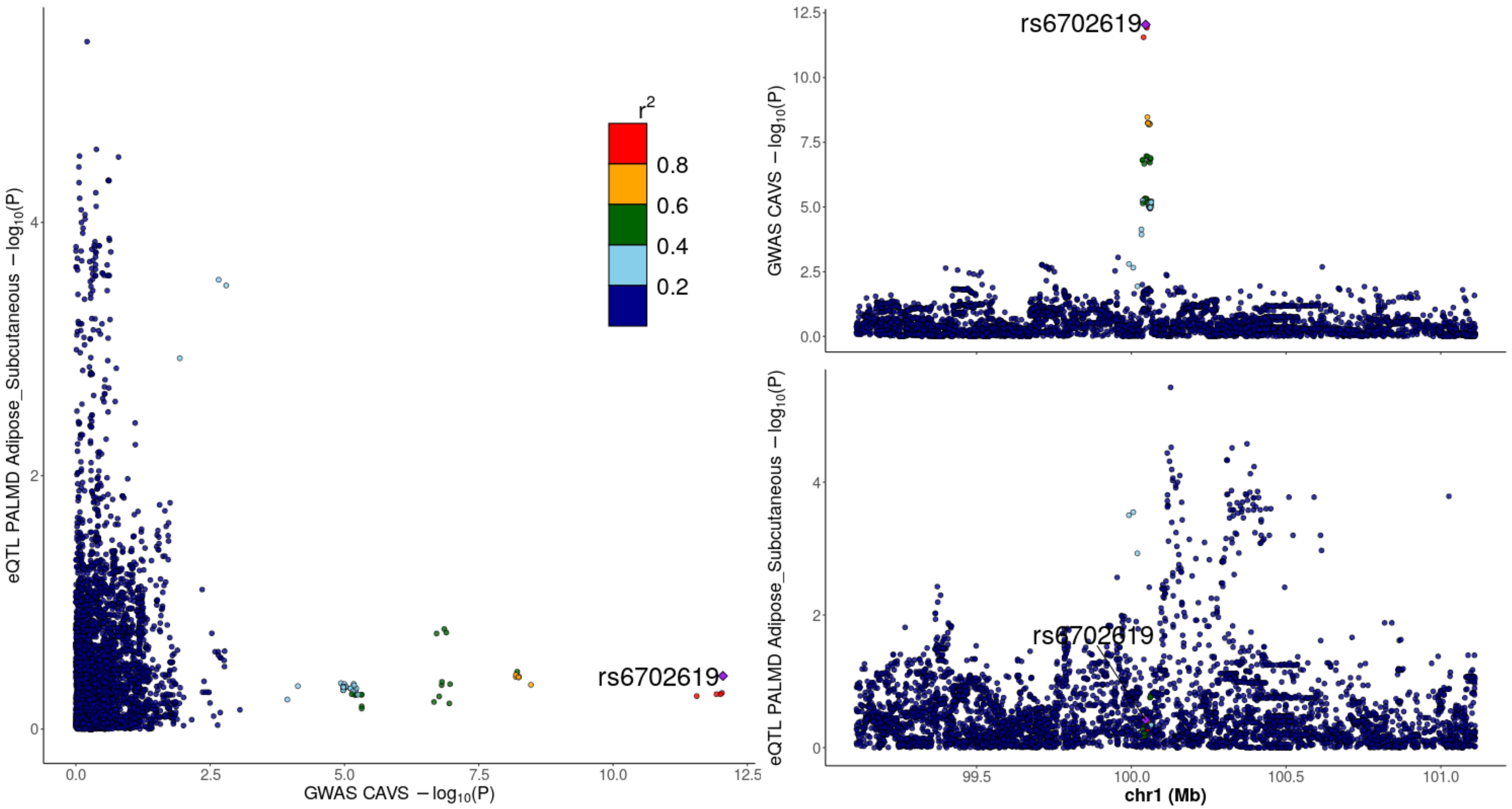

LocusCompare plots[1] showing the relationship between *PALMD* eQTL and GWAS association with CAVS for variants located within 1 Mb of *PALMD*. **A** Brain anterior cingulate cortex, colocalization PP4=3.9%; **B** Transformed fibroblasts, colocalization PP4=1.3%; **C** Gastroesophageal junction, colocalization PP4=2.0%; **D** Esophagus mucosa, colocalization PP4=1.0%; **E** Esophagus muscularis, colocalization PP4=1.1%; **F** Tibial nerve, colocalization PP4=1.0%; **G** Pancreas, colocalization PP4=2.1%; **H** Subcutaneous adipose tissue, colocalization PP4=2.3%. GWAS association was obtained from a meta-analysis of QUEBEC-CAVS and UK Biobank. The lead GWAS variant is annotated.

Supplementary Figure 4. Relationship between *PALMD* eQTL in selected tissues and GWAS association with Atrial fibrillation

A) Aortic valve

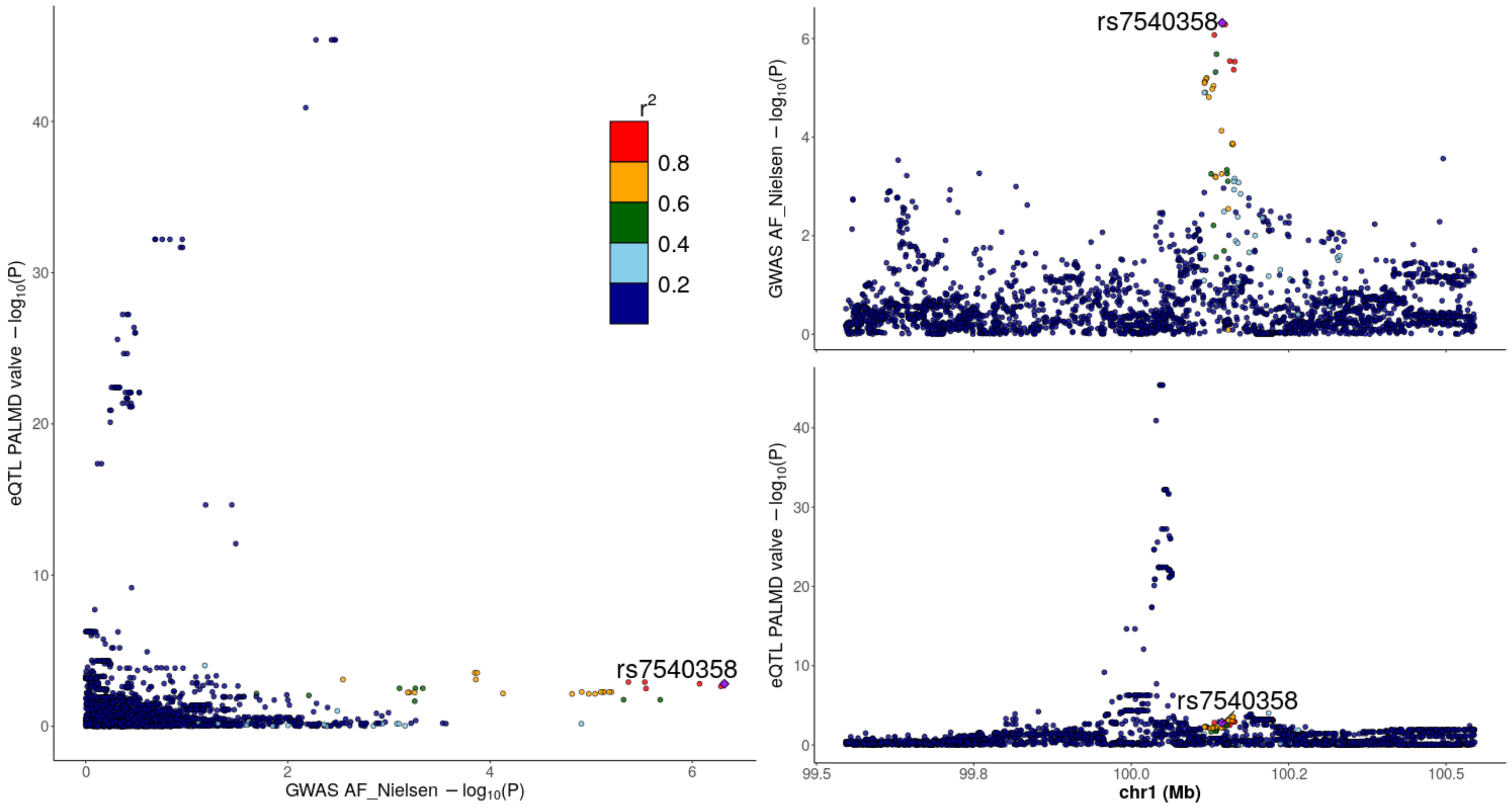

B) Brain anterior cingulate cortex

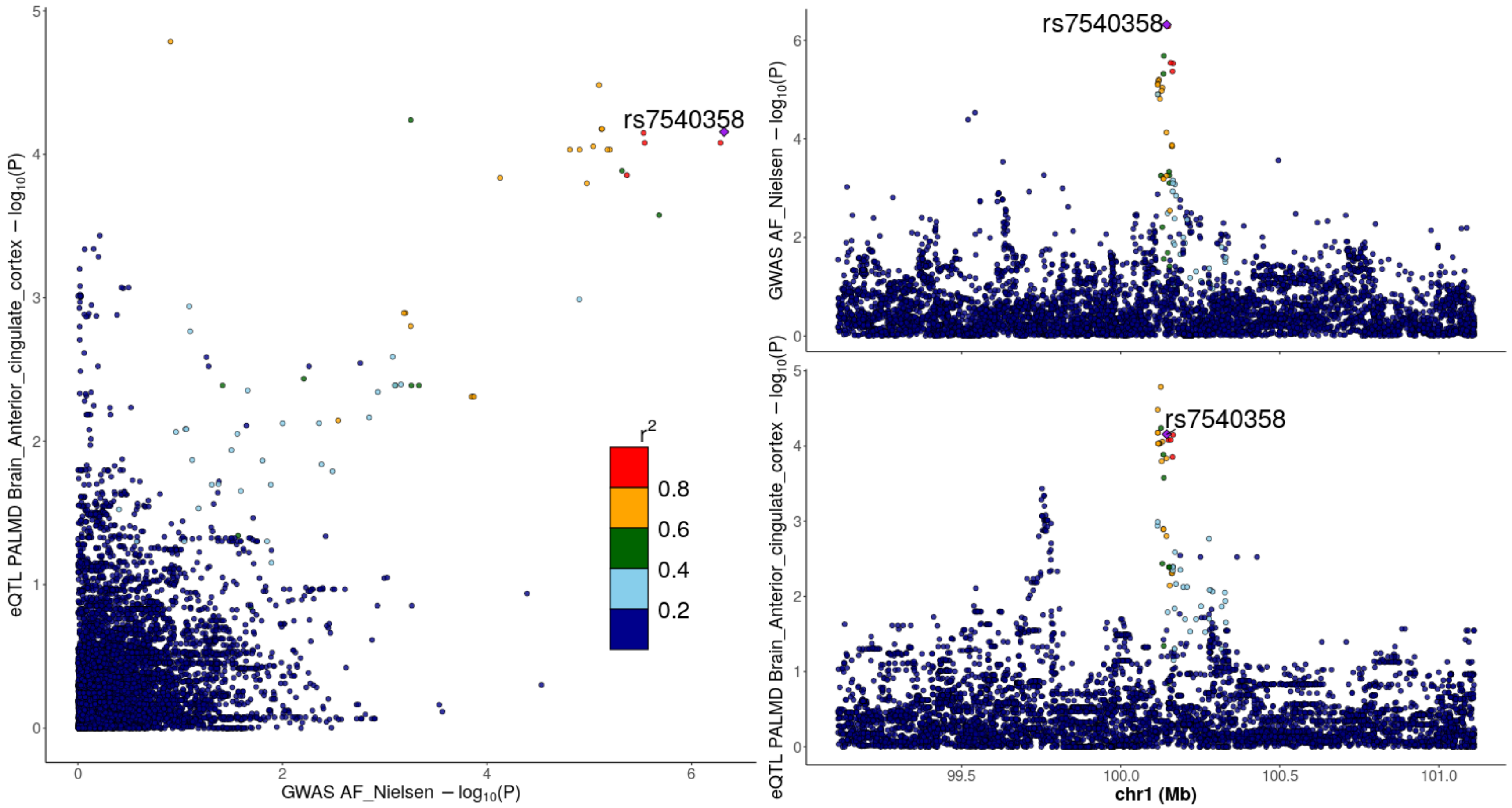

C) Esophagus muscularis

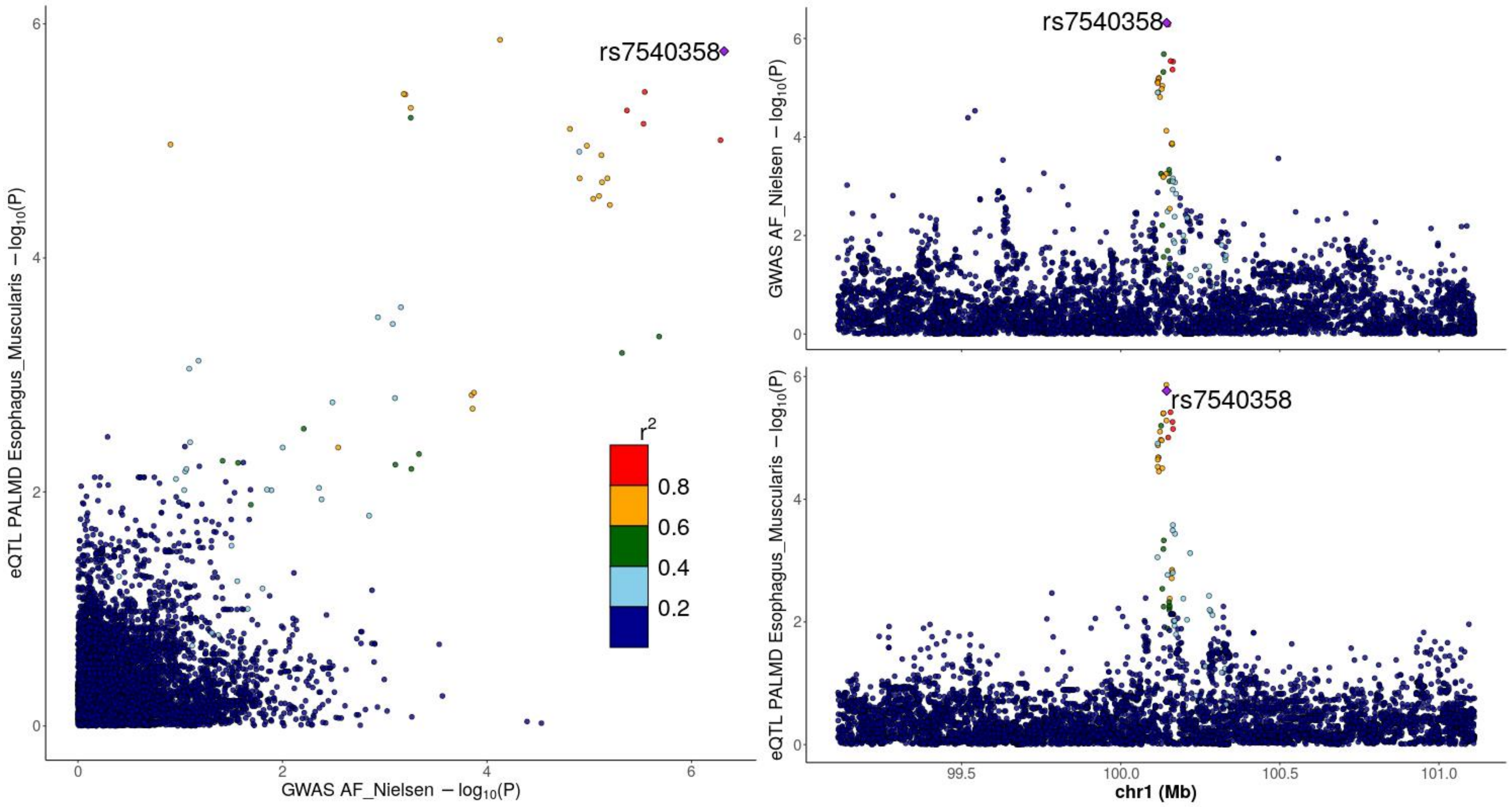

D) Tibial nerve

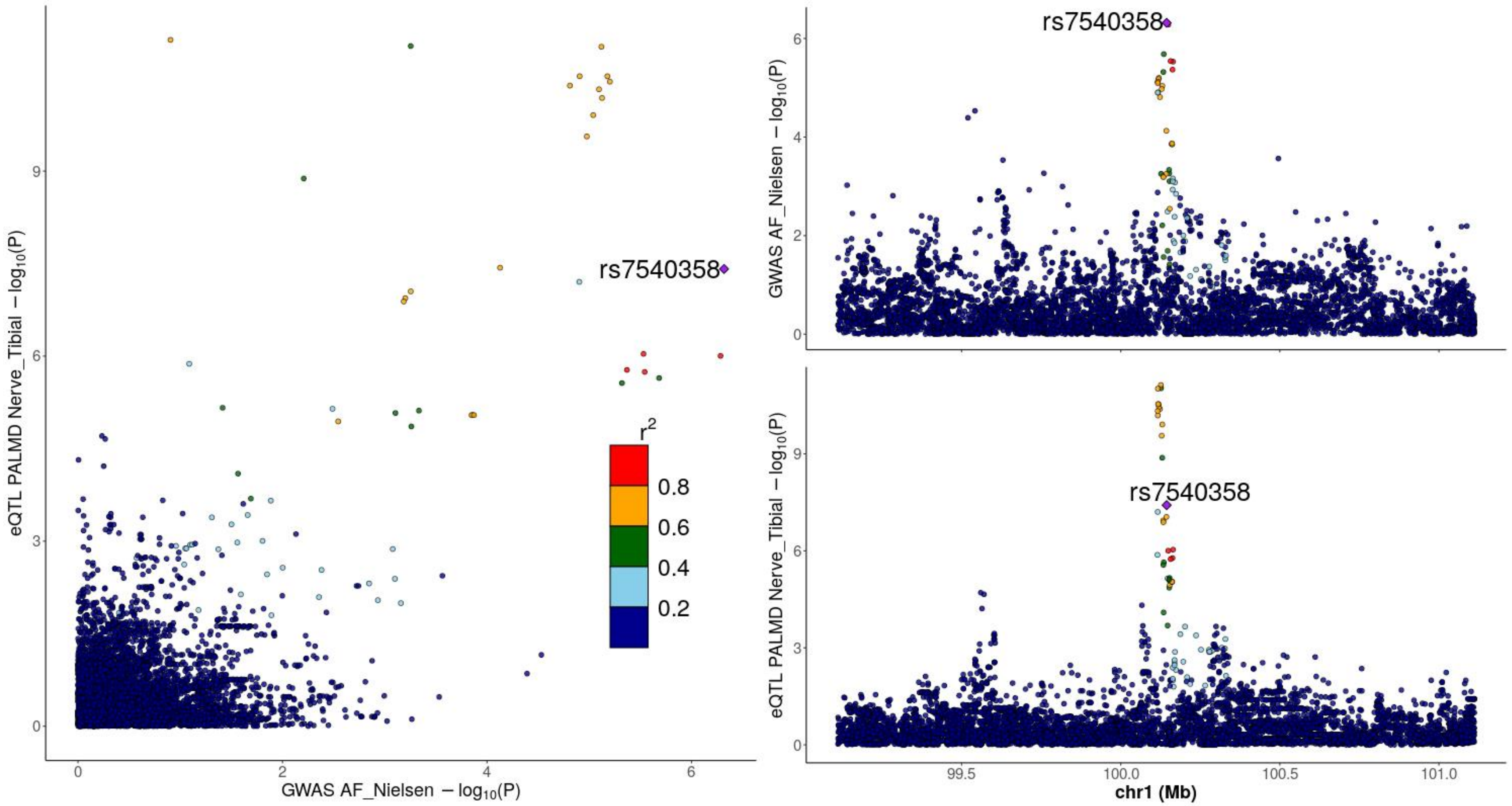

E) Subcutaneous adipose tissue

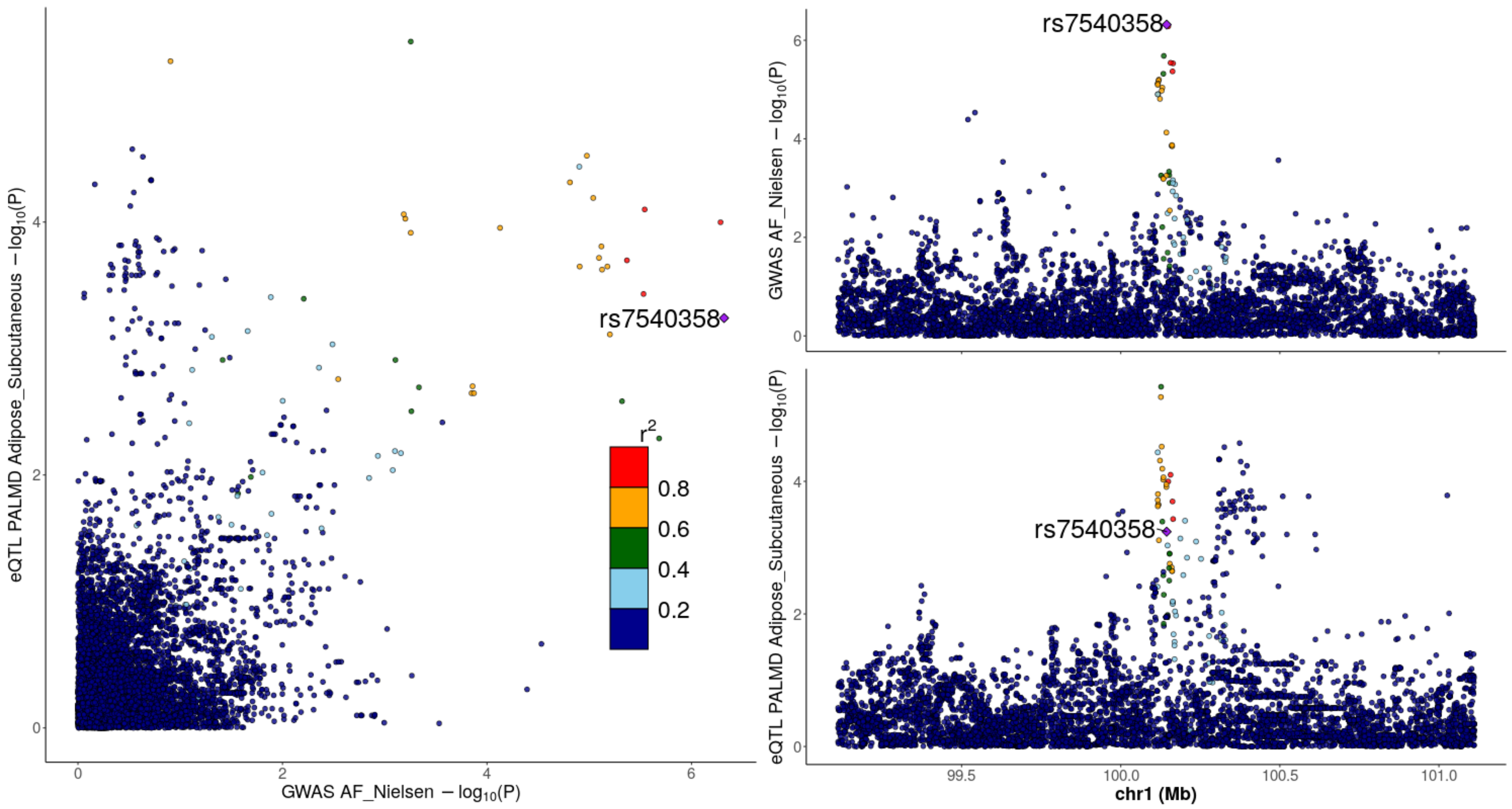

LocusCompare plots[1] showing the relationship between *PALMD* eQTL and GWAS association with Atrial fibrillation for variants located within 1 Mb of *PALMD*. **A** Aortic valve, colocalization PP4=3.2%; **B** Brain anterior cingulate cortex, colocalization PP4=61.4%; **C** Esophagus muscularis, colocalization PP4=97.0%; **D** Tibial nerve, colocalization PP4=88.6%; **E** Subcutaneous adipose tissue, colocalization PP4=77.4%. GWAS association was obtained from Nielsen et al. The lead GWAS variant is annotated.

Supplementary Tables

Supplementary Table 1. Association between *PALMD* genetically-determined expression in the aortic valve and CAVS in the UK Biobank and QUEBEC-CAVS

**Cohort**	**Sex**	**N total**	**N cases**	**N ctl**	**OR (95% OR)**	***P***
UK Biobank	Women	187369	451	186918	0.77 (0.70 - 0.85)	1.1E-07
UK Biobank	Men	163024	899	162125	0.88 (0.82 - 0.94)	0.00019
UK Biobank	All	350393	1350	349043	0.84 (0.80 - 0.89)	9.3E-10
QUEBEC-CAVS	Women	724	367	357	0.75 (0.64 - 0.87)	0.00022
QUEBEC-CAVS	Men	1302	642	660	0.89 (0.80 - 0.99)	0.037
QUEBEC-CAVS	All	2026	1009	1017	0.83 (0.76 - 0.91)	7.4E-05
Meta-analysis	Women	188093	818	187275	0.76 (0.70 - 0.83)	2.2E-10
Meta-analysis	Men	164326	1541	162785	0.88 (0.83 - 0.94)	4.0E-05
Meta-analysis	All	352419	2359	350060	0.84 (0.80 - 0.88)	1.1E-12

OR: Odds ratios for CAVS per SD increase in genetically-determined expression of *PALMD* in the aortic valve.

Supplementary Table 2. Description of the source of GWAS summary statistics for cardiovascular traits used to evaluate the impact of *PALMD* predicted expression in various tissues

Trait	Source	Study description	Sample	URL
Aortic aneurysm and/or dissection	UK Biobank Neale laboratory	UK Biobank, ICD10 code I71	361,194 Europeans, 617 cases and 360,577 controls	http://www.nealelab.is/uk-biobank
Atrial fibrillation (AF)	Nielsen et al.[2]	Meta-analysis of 6 GWAS (HUNT, deCODE, MGI, DiscovEHR, UK Biobank and AFGen)	60,620 AF cases and 970,216 controls in Europeans	http://csg.sph.umich.edu/willer/public/afib2018/
Blood pressure (DBP, SBP)	UK Biobank Neale laboratory	UK Biobank, Automated blood pressure readings, 4079, 4080 fields	317,756 and 317,754 Europeans	http://www.nealelab.is/uk-biobank
BMI	Genetic Investigation of Anthropometric Traits (GIANT)[3]	Meta-analysis of 125 GWAS and Metabochip studies	322,154 Europeans	https://www.broadinstitute.org/collaboration/giant/index.php/GIANT_consortium_data_files
CAVS	UK Biobank + QUEBEC-CAVS[4]	Meta-analysis of 2 GWAS	2359 CAVS cases and 350,060 controls in Europeans	NA
CAD	CARDIoGRAM[5]	Meta-analysis of 48 GWAS, case status defined as CAD diagnosis (i.e. MI, acute coronary syndrome, chronic stable angina or coronary stenosis of >50%)	60,801 CAD cases (approximately 70% MI) and 123,504 controls in predominantly Europeans (77%)	http://www.cardiogramplusc4d.org/
Diabetes, type 2	Diabetes Genetics Replication and Meta-Analysis (DIAGRAM)[6]	Meta-analysis of 12 GWAS, case status defined as T2D diagnosis	12,171 T2D cases and 56,862 controls in Europeans	http://diagram-consortium.org/index.html
Lipids (Total cholesterol, HDL-C, LDL-C, Triglycerides)	Global Lipids Genetics Consortium (GLGC)[7]	Meta-analysis of 60 GWAS and Metabochip studies	188,577 Europeans	http://lipidgenetics.org/

Renal function, CKD and eGFR	CKDGen[8]	Meta-analysis of 43 GWAS; CKD defined as eGFRcrea < 60 mL/min per 1.73 m^2	12,385 cases and 104,780 controls in Europeans	http://ckdgen.imbi.uni-freiburg.de/
Smoking	UK Biobank and Tobacco and Genetics (TAG) Consortium[9]	Meta-analysis of UK Biobank and TAG Consortium[10]; Smoking defined as current or previous smoker (ever smoker)	518,633 Europeans, 246,715 cases and 271,918 controls	https://www.thessgac.org/data
Stroke, all ischemic and subtypes	MEGASTROKE[11]	Meta-analysis of 29 GWAS	34,217 ischemic stroke cases (4,373 LAS; 7,193 CES; 5,386 SVS) and 406,111 controls in Europeans	http://megastroke.org/
WHR and WHR adjusted for BMI	GIANT[12]	Meta-analysis of 101 GWAS and metabochip studies	210,088 Europeans	https://www.broadinstitute.org/collaboration/giant/index.php/GIANT_consortium_data_files

References

1 Liu, B., Gloudemans, M. J., Rao, A. S., Ingelsson, E. & Montgomery, S. B. Abundant associations with gene expression complicate GWAS follow-up. *Nature genetics* **51**, 768-769, doi:10.1038/s41588-019-0404-0 (2019).

2 Nielsen, J. B. *et al.* Biobank-driven genomic discovery yields new insight into atrial fibrillation biology. *Nature genetics* **50**, 1234-1239, doi:10.1038/s41588-018-0171-3 (2018).

3 Locke, A. E. *et al.* Genetic studies of body mass index yield new insights for obesity biology. *Nature* **518**, 197-206, doi:10.1038/nature14177 (2015).

4 Theriault, S. *et al.* A transcriptome-wide association study identifies PALMD as a susceptibility gene for calcific aortic valve stenosis. *Nature communications* **9**, 988, doi:10.1038/s41467-018-03260-6 (2018).

5 Nikpay, M. *et al.* A comprehensive 1,000 Genomes-based genome-wide association meta-analysis of coronary artery disease. *Nature genetics* **47**, 1121-1130, doi:10.1038/ng.3396 (2015).

6 Morris, A. P. *et al.* Large-scale association analysis provides insights into the genetic architecture and pathophysiology of type 2 diabetes. *Nature genetics* **44**, 981-990, doi:10.1038/ng.2383 (2012).

7 Willer, C. J. *et al.* Discovery and refinement of loci associated with lipid levels. *Nature genetics* **45**, 1274-1283, doi:10.1038/ng.2797 (2013).

8 Pattaro, C. *et al.* Genetic associations at 53 loci highlight cell types and biological pathways relevant for kidney function. *Nature communications* **7**, 10023, doi:10.1038/ncomms10023 (2016).

9 Karlsson Linnér, R. *et al.* Genome-wide association analyses of risk tolerance and risky behaviors in over 1 million individuals identify hundreds of loci and shared genetic influences. *Nature genetics* **51**, 245-257, doi:10.1038/s41588-018-0309-3 (2019).

10 Genome-wide meta-analyses identify multiple loci associated with smoking behavior. *Nature genetics* **42**, 441-447, doi:10.1038/ng.571 (2010).

11 Malik, R. *et al.* Multiancestry genome-wide association study of 520,000 subjects identifies 32 loci associated with stroke and stroke subtypes. *Nature genetics* **50**, 524-537, doi:10.1038/s41588-018-0058-3 (2018).

12 Shungin, D. *et al.* New genetic loci link adipose and insulin biology to body fat distribution. *Nature* **518**, 187-196, doi:10.1038/nature14132 (2015).
