# Supplementary material for: Phenome-wide analyses establish a specific association between aortic valve PALMD expression and calcific aortic valve stenosis
Source: Commun Biol. 2020 Aug 28;3:477. doi: 10.1038/s42003-020-01210-x (PMC7455695; doi:10.1038/s42003-020-01210-x)
Supplement: Supplementary file 2 — Description of Additional Supplementary Files [file 42003_2020_1210_MOESM2_ESM.pdf]

Description of Additional Supplementary Files

Supplementary Data 1. Variants in the models used to predict *PALMD* expression in 9 tissues. Table listing the variants and their weights in models used to predict *PALMD* expression in 9 tissues. Chr: Chromosome; Pos: Position (GRCh37).

Supplementary Data 2. Description of the 852 phenotypes used in the phenome-wide analyses in the UK Biobank. ICD: International Classification of Diseases 10th Revision; Clinical: phenotypes obtained from participants visits (questionnaire or examination); OPCS: Office of Population Censuses and Surveys Classification of Surgical Operations and Procedures, 4th Revision.

Supplementary Data 3. Results of the Phenome-wide association study for *PALMD* genetically-determined expression in the aortic valve. *P*_FDR: p-value adjusted using false discovery rate (Benjamini & Hochberg) method; *P*_BY: p-value adjusted using Benjamini & Yekutieli method; *P*_Bonf: p-value adjusted using Bonferroni correction; ICD: International Classification of Diseases 10th Revision; Clinical: phenotypes obtained from participants visits (questionnaire or examination); OPCS: Office of Population Censuses and Surveys Classification of Surgical Operations and Procedures, 4th Revision; NCI: non-cancer illness code (self-reported).

Supplementary Data 4. Results of the Phenome-wide association study for rs6702619-G. *P*_FDR: p-value adjusted using false discovery rate (Benjamini & Hochberg) method; *P*_BY: p-value adjusted using Benjamini & Yekutieli method; *P*_Bonf: p-value adjusted using Bonferroni correction; ICD: International Classification of Diseases 10th Revision; Clinical: phenotypes obtained from participants visits (questionnaire or examination); OPCS: Office of Population Censuses and Surveys Classification of Surgical Operations and Procedures, 4th Revision; NCI: non-cancer illness code (self-reported).

Supplementary Data 5. eQTL with *P*<0.001 for rs6702619-G in 49 tissues (cis and trans). Table showing all eQTL with *P*<0.001 for the G allele at rs6702619 in the aortic valve and 48 other tissues, including cis and trans results. Distance: distance between rs6702619 and the transcription start site of the gene (number of bases); *P*_FDR: p-value adjusted using false discovery rate (Benjamini & Hochberg) method; *P*_BY: p-value adjusted using Benjamini & Yekutieli method; *P*_Bonf: p-value adjusted using Bonferroni correction;

Supplementary Data 6. Results of the Phenome-wide association study for *PALMD* genetically-determined expression in brain anterior cingulate cortex. *P*_FDR: p-value adjusted using false discovery rate (Benjamini & Hochberg) method; *P*_BY: p-value adjusted using Benjamini & Yekutieli method; *P*_Bonf: p-value adjusted using Bonferroni correction; ICD: International Classification of Diseases 10th Revision; Clinical: phenotypes obtained from participants visits (questionnaire or examination); OPCS: Office of Population Censuses and

Surveys Classification of Surgical Operations and Procedures, 4th Revision; NCI: non-cancer illness code (self-reported).

Supplementary Data 7. Results of the Phenome-wide association study for *PALMD* genetically-determined expression in transformed fibroblasts. *P*_FDR: p-value adjusted using false discovery rate (Benjamini & Hochberg) method; *P*_BY: p-value adjusted using Benjamini & Yekutieli method; *P*_Bonf: p-value adjusted using Bonferroni correction; ICD: International Classification of Diseases 10th Revision; Clinical: phenotypes obtained from participants visits (questionnaire or examination); OPCS: Office of Population Censuses and Surveys Classification of Surgical Operations and Procedures, 4th Revision; NCI: non-cancer illness code (self-reported).

Supplementary Data 8. Results of the Phenome-wide association study for *PALMD* genetically-determined expression in gastroesophageal junction. *P*_FDR: p-value adjusted using false discovery rate (Benjamini & Hochberg) method; *P*_BY: p-value adjusted using Benjamini & Yekutieli method; *P*_Bonf: p-value adjusted using Bonferroni correction; ICD: International Classification of Diseases 10th Revision; Clinical: phenotypes obtained from participants visits (questionnaire or examination); OPCS: Office of Population Censuses and Surveys Classification of Surgical Operations and Procedures, 4th Revision; NCI: non-cancer illness code (self-reported).

Supplementary Data 9. Results of the Phenome-wide association study for *PALMD* genetically-determined expression in esophagus mucosa. *P*_FDR: p-value adjusted using false discovery rate (Benjamini & Hochberg) method; *P*_BY: p-value adjusted using Benjamini & Yekutieli method; *P*_Bonf: p-value adjusted using Bonferroni correction; ICD: International Classification of Diseases 10th Revision; Clinical: phenotypes obtained from participants visits (questionnaire or examination); OPCS: Office of Population Censuses and Surveys Classification of Surgical Operations and Procedures, 4th Revision; NCI: non-cancer illness code (self-reported).

Supplementary Data 10. Results of the Phenome-wide association study for *PALMD* genetically-determined expression in esophagus muscularis. *P*_FDR: p-value adjusted using false discovery rate (Benjamini & Hochberg) method; *P*_BY: p-value adjusted using Benjamini & Yekutieli method; *P*_Bonf: p-value adjusted using Bonferroni correction; ICD: International Classification of Diseases 10th Revision; Clinical: phenotypes obtained from participants visits (questionnaire or examination); OPCS: Office of Population Censuses and Surveys Classification of Surgical Operations and Procedures, 4th Revision; NCI: non-cancer illness code (self-reported).

Supplementary Data 11. Results of the Phenome-wide association study for *PALMD* genetically-determined expression in tibial nerve. *P*_FDR: p-value adjusted using false discovery rate (Benjamini & Hochberg) method; *P*_BY: p-value adjusted using Benjamini & Yekutieli method; *P*_Bonf: p-value adjusted using Bonferroni correction; ICD: International Classification of Diseases 10th Revision; Clinical: phenotypes obtained from participants visits (questionnaire or examination); OPCS: Office of Population Censuses and Surveys Classification of Surgical Operations and Procedures, 4th Revision; NCI: non-cancer illness code (self-reported).

Supplementary Data 12. Results of the Phenome-wide association study for *PALMD* genetically-determined expression in pancreas. *P*_FDR: p-value adjusted using false discovery rate (Benjamini & Hochberg) method; *P*_BY: p-value adjusted using Benjamini & Yekutieli method; *P*_Bonf: p-value adjusted using Bonferroni correction; ICD: International Classification of Diseases 10th Revision; Clinical: phenotypes obtained from participants visits (questionnaire or examination); OPCS: Office of Population Censuses and Surveys Classification of Surgical Operations and Procedures, 4th Revision; NCI: non-cancer illness code (self-reported).

Supplementary Data 13. Results of the Phenome-wide association study for *PALMD* genetically-determined expression in subcutaneous adipose tissue. *P*_FDR: p-value adjusted using false discovery rate (Benjamini & Hochberg) method; *P*_BY: p-value adjusted using Benjamini & Yekutieli method; *P*_Bonf: p-value adjusted using Bonferroni correction; ICD: International Classification of Diseases 10th Revision; Clinical: phenotypes obtained from participants visits (questionnaire or examination); OPCS: Office of Population Censuses and Surveys Classification of Surgical Operations and Procedures, 4th Revision; NCI: non-cancer illness code (self-reported).

Supplementary Data 14. Association between *PALMD* genetically-determined expression in 9 tissues and 21 cardiovascular phenotypes using S-PrediXcan. Source: GWAS meta-analysis used as described in **Supplementary Table 2**; *P*_FDR: p-value adjusted using false discovery rate (Benjamini & Hochberg) method; *P*_BY: p-value adjusted using Benjamini & Yekutieli method; *P*_Bonf: p-value adjusted using Bonferroni correction.
