# Supplementary material for: Phenome-wide analyses establish a specific association between aortic valve PALMD expression and calcific aortic valve stenosis
Source: Commun Biol. 2020 Aug 28;3:477. doi: 10.1038/s42003-020-01210-x (PMC7455695; doi:10.1038/s42003-020-01210-x)
Supplement: Supplementary file 4 — Reporting Summary [file 42003_2020_1210_MOESM4_ESM.pdf]

nature research

Corresponding author(s): Sébastien Thériault

Last updated by author(s): Jul 15, 2020

# Reporting Summary

Nature Research wishes to improve the reproducibility of the work that we publish. This form provides structure for consistency and transparency in reporting. For further information on Nature Research policies, see Authors & Referees and the Editorial Policy Checklist .

## Statistics

For all statistical analyses, confirm that the following items are present in the figure legend, table legend, main text, or Methods section.

| n/a | Confirmed | |
|---|---|---|
| ☐ | ☒ | The exact sample size ($n$) for each experimental group/condition, given as a discrete number and unit of measurement |
| ☐ | ☒ | A statement on whether measurements were taken from distinct samples or whether the same sample was measured repeatedly |
| ☐ | ☒ | The statistical test(s) used AND whether they are one- or two-sided<br>*Only common tests should be described solely by name; describe more complex techniques in the Methods section.* |
| ☐ | ☒ | A description of all covariates tested |
| ☐ | ☒ | A description of any assumptions or corrections, such as tests of normality and adjustment for multiple comparisons |
| ☐ | ☒ | A full description of the statistical parameters including central tendency (e.g. means) or other basic estimates (e.g. regression coefficient) AND variation (e.g. standard deviation) or associated estimates of uncertainty (e.g. confidence intervals) |
| ☐ | ☒ | For null hypothesis testing, the test statistic (e.g. $F$, $t$, $r$) with confidence intervals, effect sizes, degrees of freedom and $P$ value noted<br>*Give P values as exact values whenever suitable.* |
| ☒ | ☐ | For Bayesian analysis, information on the choice of priors and Markov chain Monte Carlo settings |
| ☒ | ☐ | For hierarchical and complex designs, identification of the appropriate level for tests and full reporting of outcomes |
| ☐ | ☒ | Estimates of effect sizes (e.g. Cohen's $d$, Pearson's $r$), indicating how they were calculated |

*Our web collection on statistics for biologists contains articles on many of the points above.*

## Software and code

Policy information about availability of computer code

| | |
|---|---|
| Data collection | No software was used for data collection. |
| Data analysis | Analyses were performed using R version 3.5.1, specific packages are mentioned in the manuscript (rmeta, PheWAS, LocusCompareR, coloc).<br>The PrediXcan, S-PrediXcan, SNPTEST and QTLtools softwares were used (references in the manuscript). |

For manuscripts utilizing custom algorithms or software that are central to the research but not yet described in published literature, software must be made available to editors/reviewers. We strongly encourage code deposition in a community repository (e.g. GitHub). See the Nature Research guidelines for submitting code & software for further information.

## Data

Policy information about availability of data

All manuscripts must include a data availability statement. This statement should provide the following information, where applicable:

- Accession codes, unique identifiers, or web links for publicly available datasets
- A list of figures that have associated raw data
- A description of any restrictions on data availability

The UK Biobank data is publicly available following an application request. The microarray gene expression data set on human aortic valves was deposited in Gene Expression Omnibus with accession number GSE102249. Transcriptome prediction models are available in the PredictDB Data Repository (http://predictdb.org/). GTEx Project data are available at https://www.gtexportal.org/home/ and on dbGaP (accession phs000424.v8.p2). Links for the publicly available summary statistics used in this work are available in Supplementary Table 2.

# Field-specific reporting

Please select the one below that is the best fit for your research. If you are not sure, read the appropriate sections before making your selection.

- [x] Life sciences
- [ ] Behavioural & social sciences
- [ ] Ecological, evolutionary & environmental sciences

For a reference copy of the document with all sections, see nature.com/documents/nr-reporting-summary-flat.pdf

# Life sciences study design

All studies must disclose on these points even when the disclosure is negative.

| | |
|---|---|
| Sample size | All available data from the studied cohorts and databases (UK Biobank, QUEBEC-CAVS, GTEx Project) were used to maximize power. |
| Data exclusions | No data were excluded, except for pre-established quality control purposes (see description in the Methods section). |
| Replication | Data from two cohorts were analyzed when possible to confirm the findings (UK Biobank and QUEBEC-CAVS). Different approaches were used to investigate effects on cardiovascular phenotypes (PheWAS in UK Biobank and S-PrediXcan analyses using publicly available summary statistics). |
| Randomization | Not relevant to the current study, analyses performed on biobank data. |
| Blinding | Not relevant to the current study, analyses performed on biobank data. |

# Reporting for specific materials, systems and methods

We require information from authors about some types of materials, experimental systems and methods used in many studies. Here, indicate whether each material, system or method listed is relevant to your study. If you are not sure if a list item applies to your research, read the appropriate section before selecting a response.

## Materials & experimental systems

| n/a | Involved in the study |
|---|---|
| x | Antibodies |
| x | Eukaryotic cell lines |
| x | Palaeontology |
| x | Animals and other organisms |
| | x Human research participants |
| x | Clinical data |

## Methods

| n/a | Involved in the study |
|---|---|
| x | ChIP-seq |
| x | Flow cytometry |
| x | MRI-based neuroimaging |

## Human research participants

Policy information about studies involving human research participants

| | |
|---|---|
| Population characteristics | The UK Biobank dataset included 353,378 European individuals (mean age 57 years, 54% female). The QUEBEC-CAVS dataset included 2,026 indviduals (mean age 72 years, 64% male). Detailed population characteristics according to CAVS status have been published before (PMID 32141789). |
| Recruitment | Recruitment for the UK Biobank (prospective population study) and QUEBEC-CAVS (case-control study for CAVS) have been published before (PMID 25826379 and 29511167). |
| Ethics oversight | The study was approved by the ethics committee of the Institut universitaire de cardiologie et de pneumologie de Québec-Université Laval. |

Note that full information on the approval of the study protocol must also be provided in the manuscript.
